# Extracorporeal membrane oxygenation for acute respiratory distress syndrome in burn patients: a case series and literature update

**DOI:** 10.1186/s41038-019-0166-z

**Published:** 2019-11-01

**Authors:** Mehran Dadras, Johannes M. Wagner, Christoph Wallner, Julika Huber, Dirk Buchwald, Justus Strauch, Kamran Harati, Nicolai Kapalschinski, Björn Behr, Marcus Lehnhardt

**Affiliations:** 1 Department of Plastic Surgery, BG University Hospital Bergmannsheil, Bürkle de la Camp-Platz 1, 44789 Bochum, Germany; 2 Department of Cardiothoracic Surgery, BG University Hospital Bergmannsheil, Bürkle de la Camp-Platz 1, 44789 Bochum, Germany

**Keywords:** Extracorporeal membrane oxygenation, Acute respiratory distress syndrome, Burns, Inhalation injury, ECMO, ARDS

## Abstract

**Background:**

Acute respiratory distress syndrome (ARDS) has a reported incidence of 34–43% in ventilated burn patients and is associated with a mortality of 59% in the severe form. The use and experience with extracorporeal membrane oxygenation (ECMO) in burn patients developing ARDS are still limited. We present our results and discuss the significance of ECMO in treating burn patients.

**Methods:**

A retrospective analysis of burn patients treated with ECMO for ARDS between January 2017 and January 2019 was performed. Demographic, clinical, and outcome data were collected and analyzed.

**Results:**

Eight burn patients were treated at our institution with ECMO in the designated time period. Of these, all but one patient had inhalation injury, burn percentage of TBSA was 37 ± 23%, ABSI score was 8.4 ± 2, and R-Baux-score was 98 ± 21. Seven patients developed severe ARDS and one patient moderate ARDS according to the Berlin classification with a PaO_2_/FiO_2_ ratio upon initiation of ECMO therapy of 62 ± 22 mmHg. ECMO duration was 388 ± 283 h. Three patients died from severe sepsis while five patients survived to hospital discharge.

**Conclusions:**

ECMO is a viable therapy option in burn patients developing severe ARDS and can contribute to survival rates similar to ECMO therapy in non-burn-associated severe ARDS. Consequently, patients with severe respiratory insufficiency with unsuccessful conventional treatment and suspected worsening should be transferred to burn units with the possibility of ECMO treatment to improve outcome.

## Background

In patients with severe burns and smoke inhalation injury, the development of acute respiratory distress syndrome (ARDS) poses a tremendous clinical challenge.

The incidence of ARDS in burn and inhalation injury patients requiring mechanical ventilation is reported in the range of 34–43% with mortality rates reaching 59.7% for severe ARDS following Berlin criteria [1, 2].

The etiology of an evolving ARDS can be multifactorial. Smoke inhalation injury, fluid shifts after burn injury and resuscitation, systemic inflammation due to thermal injury, or secondary pneumonia can all be factors that promote development of ARDS. In general, it is accepted that ARDS therapy should consist of fluid restriction, protective ventilation with low tidal volume and high positive end-expiratory pressure (PEEP), prone positioning, and neuromuscular blockade. Adjunctive therapies consist of inverted ratio ventilation, high frequency ventilation, and inhalative vasodilators like nitric oxide (NO) to reduce pulmonary hypertension, although evidence is limited for these measures [3, 4].

Ultimately, in patients with therapy refractory severe ARDS, the application of extracorporeal membrane oxygenation (ECMO) represents a treatment option.

ECMO uses large-diameter cannulae to drain venous blood to an oxygenator device; depending on the two most basic settings, the oxygenated and decarboxylated blood is then reinfused via a venous cannula (veno-venous ECMO, V-V-ECMO) or via an arterial cannula (veno-arterial ECMO, V-A-ECMO). While the latter offers full circulatory support, veno-venous ECMO is used as lung support for ARDS treatment and performs extracorporeal gas exchange.

The guidelines of the German Society of Anesthesiology and Intensive Care Medicine and British Faculty of Intensive Care Medicine recommend consideration of ECMO in severe ARDS refractory to conventional therapy while the guidelines of the American Thoracic Society demand further evidence before a statement for or against the use of ECMO [5–7]. However, none of these guidelines include the latest randomized controlled trial published in July 2018 [8].

The literature on the use of ECMO in patients with burn and inhalation injury is limited. Retrospective inquiry at the Extracorporeal Life Support Organization (ELSO) international registry resulted in 58 patients from 1999 to 2015 with a hospital mortality rate of 57% [9]. Soussi et al. in 2016 reported a 28% 90-day survival rate and 9% hospital survival rate in 11 burn patients receiving ECMO therapy [10]. Recently, Ainsworth et al. reported a hospital mortality of 54% in 11 adult burn patients receiving ECMO and Eldredge et al. reported a hospital mortality of 12.5% in a cohort of eight mainly pediatric burn patients [11, 12].

At our institution, a burn center and a heart surgery department with ECMO center are existent. We present our data on burn patients receiving ECMO therapy and emphasize the remarkable clinical course of one patient.

## Methods

All consecutive patients with burn injury who were treated in BG University Hospital Bergmannsheil from January 2017 until January 2019 and received ECMO treatment were included.

Retrospectively, demographics of the patients, clinical data, their course of treatment including complications, and outcome were collected using the medical records.

Burn percentage of the total body surface area (TBSA) and depth of burn were assessed by clinical examination using Lund-Browder charts. All patients received fiberoptic bronchoscopy upon admission to diagnose smoke inhalation injury. Bronchoscopies in ARDS patients were not performed routinely thereafter. The Berlin definition of ARDS was used to diagnose ARDS and assess severity [13]. The Sequential Organ Failure Assessment (SOFA) score and the Simplified Acute Physiology Score (SAPS) II were used to assess organ dysfunction and severity [14, 15].

Crystalloid fluid resuscitation in patients with > 20% burns was started according to the Parkland formula and titrated to an ideal urine output of 0.5–1 ml/kg/h as the target value. Serum albumin target range was 3–3.5 g/dl and serum sodium was held in the reference range of 135–145 mmol/l. Mean arterial pressure of at least 65 mmHg was aimed for.

Cardiohelp (Maquet, Rastatt, Germany) was the ECMO device used in all patients. ECMO cannulation was performed by anesthesiologists or cardiothoracic surgeons under ultrasound guidance.

Continuous variables are expressed as mean ± standard deviation (SD) or median and range and categorical data as frequencies and percentages.

## Results

A total of eight patients were included with the data presented in Table 1. Of these, two were female and six were male, and the median age was 48 years.

**Table 1:** Study population with data on each patient

	Patient	1	2	3	4	5	6	7	8
Sex		M	F	F	M	M	M	M	M
Age (years)		52	45	34	21	69	35	54	58
Injury									
	Burn percentage of TBSA	65	26	15	75	45	40	15	17
	Inhalation injury	No	Yes	Yes	Yes	Yes	Yes	Yes	Yes
	ABSI score	9	9	6	12	10	7	7	7
	R-Baux score	117	88	66	113	131	92	86	92
ARDS									
	Etiology: Immediate trauma	Yes	Yes	Yes	Yes	No	Yes	No	No
	PaO_2_/FiO_2_ (mmHg)	59	42	44	50	63	55	110	70
	Murray score	3.7	3.8	3.5	3.5	3.7	3.3	3.3	3.3
Measures prior to ECMO									
	Prone positioning	No	Yes	Yes	Yes	No	Yes	Yes	Yes
	Nitric oxide inhalation	No	No	No	Yes	No	No	No	No
	Recruitment maneuver	Yes	Yes	Yes	Yes	Yes	Yes	Yes	Yes
	Neuromuscular blockade	Yes	Yes	Yes	Yes	Yes	Yes	Yes	Yes
Characteristics at ECMO onset									
	SOFA score	19	13	9	13	19	10	10	6
	SAPS II score	49	48	27	36	57	25	30	32
	Minute ventilation (l)	12.1	8.5	7	10.1	8	10	9.9	12
	Tidal volume (ml)	550	599	320	430	400	520	380	650
	Respiratory rate (/min)	22	15	22	24	22	18	26	18
	PEEP (mmHg)	13	20	11	11	12	15	14	12
	Plateau pressure (mmHg)	42	40	35	35	35	35	30	30
	Compliance (ml/cm H_2_O)	14	22	9.8	13.2	12.8	19.1	17.4	26.5
	Arterial pCO2 (mmHg)	40	85	78	60	80	66	115	55
	Arterial pH	7.38	7.23	7.24	7.28	7.3	7.3	7.07	7.43
	Arterial lactate (mmol/l)	19.6	9.3	1.9	4.4	9.6	1.1	0.9	0.6
	Serum bilirubin (mg/dl)	4.4	0.9	0.7	1.1	8.9	0.6	1.2	0.8
	Serum creatinine (mg/dl)	1.8	1.4	0.8	1.9	3.4	0.9	1	0.9
Outcome									
	ECMO duration (h)	288	528	480	984	48	240	288	248
	Hospital stay (days)	15	22	30	178	21	64	33	80
	ICU stay (days)	15	22	20	178	21	34	33	80
	Renal replacement (days)	15	0	4	76	4	0	0	0
	Mechanic ventilation (days)	15	22	16	120	21	26	24	50
	Survival to discharge	No	No	Yes	Yes	No	Yes	Yes	Yes
	Cause of death	Sepsis	Sepsis	–	–	Sepsis	–	–	–

*TBSA* total body surface area, *ABSI* Abbreviated Burn Severity Index, *SOFA* Sequential Organ Failure Assessment, *SAPS II* Simplified Acute Physiology Score II, *PEEP* positive end-expiratory pressure, *ICU* intensive care unit, *ARDS* acute respiratory distress syndrome, *ECMO* extracorporeal membrane oxygenation, *M* male, *F* famale

### Burn severity

Burned TBSA was 37 ± 23%, and all but one patient suffered smoke inhalation injury, which was confirmed bronchoscopically.

The Abbreviated Burn Severity Index (ABSI) score of the patients ranged from 6 to 12 with a median of 8 and the R-Baux score from 66 to 131 with a median of 92. Four patients had acute kidney failure and required continuous renal replacement therapy. Patient 4 had an additional cytokine filter installed due to sepsis.

Four patients needed escharotomy due to deep circular burns and two patients developed abdominal compartment syndrome with the need for laparotomy.

### Development of ARDS

ARDS occurred as a direct complication of burn/inhalation trauma (within 6 days) in five patients, while the other three patients developed secondary ARDS due to respiratory infection 10, 14, and 19 days after burn injury. All but one patient fulfilled the Berlin diagnostic criteria for severe ARDS while in one patient, only moderate ARDS could be diagnosed with a leading hypercapnia and arterial pH of 7.07 in this patient.

Prone positioning was performed in six patients and NO-inhalative therapy in one patient before initiation of ECMO. Prone positioning was not performed in two patients due to open abdomen after decompression of abdominal compartment.

### Initiation of ECMO treatment

ECMO was initiated < 24 h after diagnosis of severe ARDS in all patients.

**Fig. 1:**
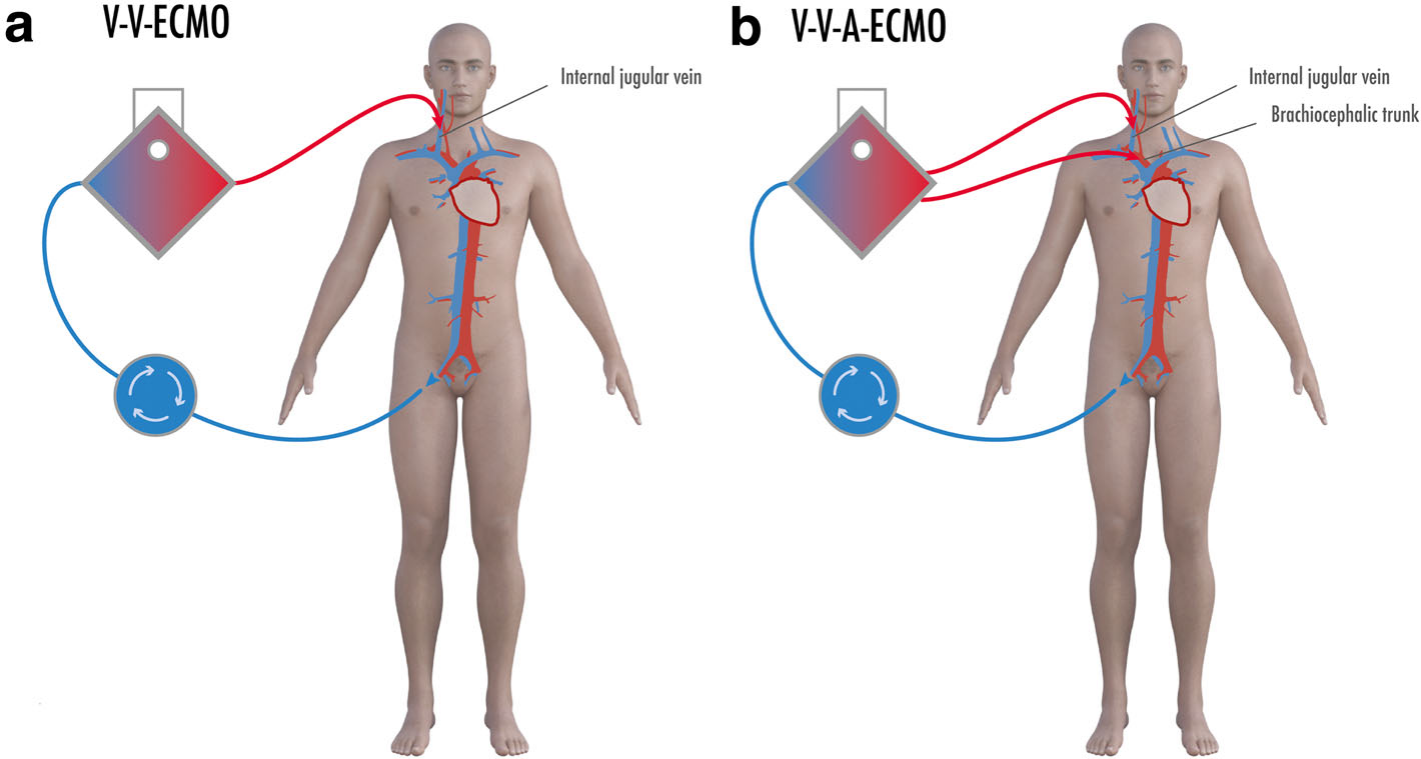
Extracorporeal membrane oxygenation (ECMO) configurations. **a** Typical two-cannula veno-venous (V-V-) ECMO, drainage of blood from femoral vein, and reinfusion via internal jugular vein after oxygenation and decarboxylation. **b** veno-veno-arterial (V-V-A)-ECMO with additional arterial infusion cannula in the brachiocephalic trunk used in patient 4 to ensure brain oxygenation

All patients primarily received veno-venous ECMO. In five patients, a two-cannula approach via right internal jugular vein and femoral vein was chosen (Fig. 1a) while in three patients, a dual lumen Avalon cannula (Getinge, Getinge, Sweden) via the right internal jugular vein was utilized. In patient 4, secondarily, a veno-veno-arterial (V-V-A-) ECMO setup was used with an additional arterial outflow cannula in the brachiocephalic trunk (Fig. 1b). This case is presented in detail further below.

Mean PaO2/FiO2 ratio upon initiation of ECMO therapy was 62 ± 22 mmHg while PaCO_2_ was 72 ± 23 mmHg and arterial pH was 7.28 ± 0.11. PEEP was 13 ± 3.2 cm H_2_O, driving pressure 21.7 ± 4 cm H_2_O, and Murray lung injury score was 3.5 ± 0.2 upon onset of ECMO therapy. Mean SOFA severity of illness score at onset of ECMO treatment was 12.4 ± 4.7, for which a roughly 50% mortality can be estimated based on previous literature.[16] Mean SAPSII at onset of ECMO treatment was 38 ± 12, predicting a mortality of 25% [15].

### ECMO treatment

**Fig. 2:**
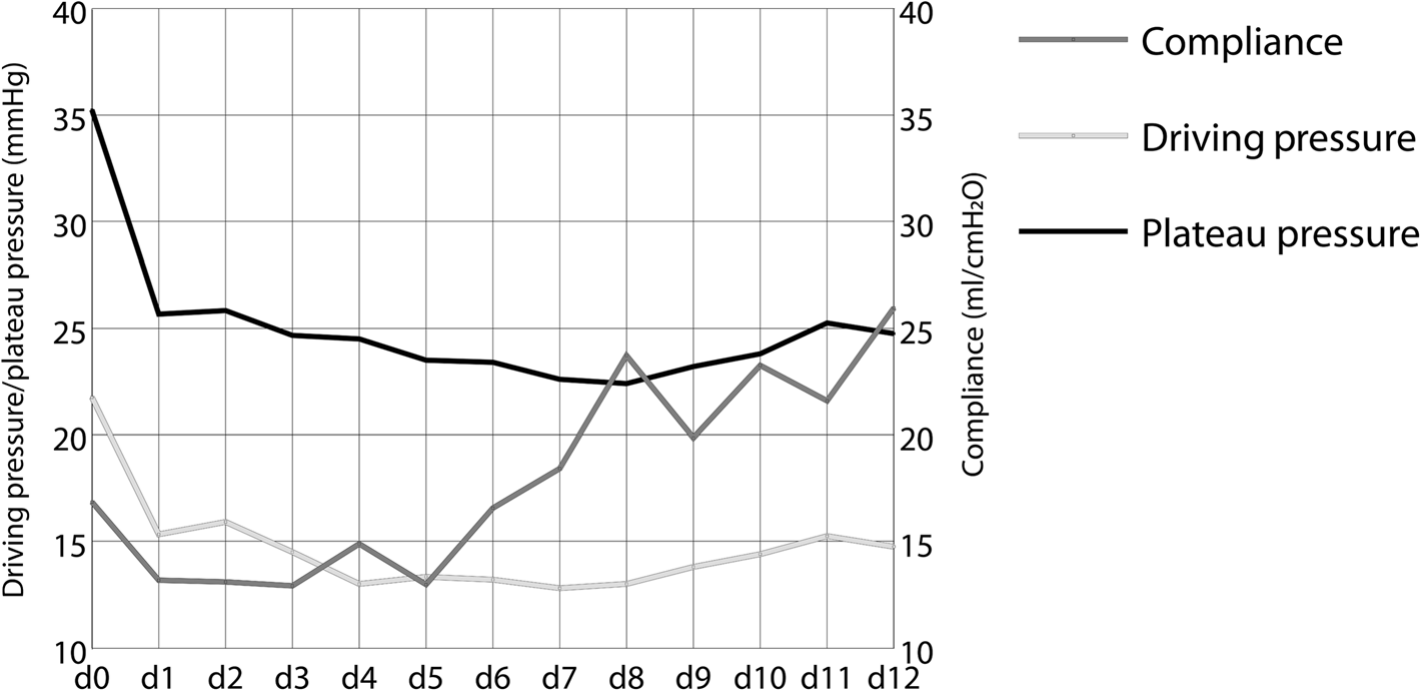
Mean plateau pressure, driving pressure, and compliance at onset of extracorporeal membrane oxygenation (ECMO) (day 0) and 12 consecutive days (*n* = 8)

**Fig. 3:**
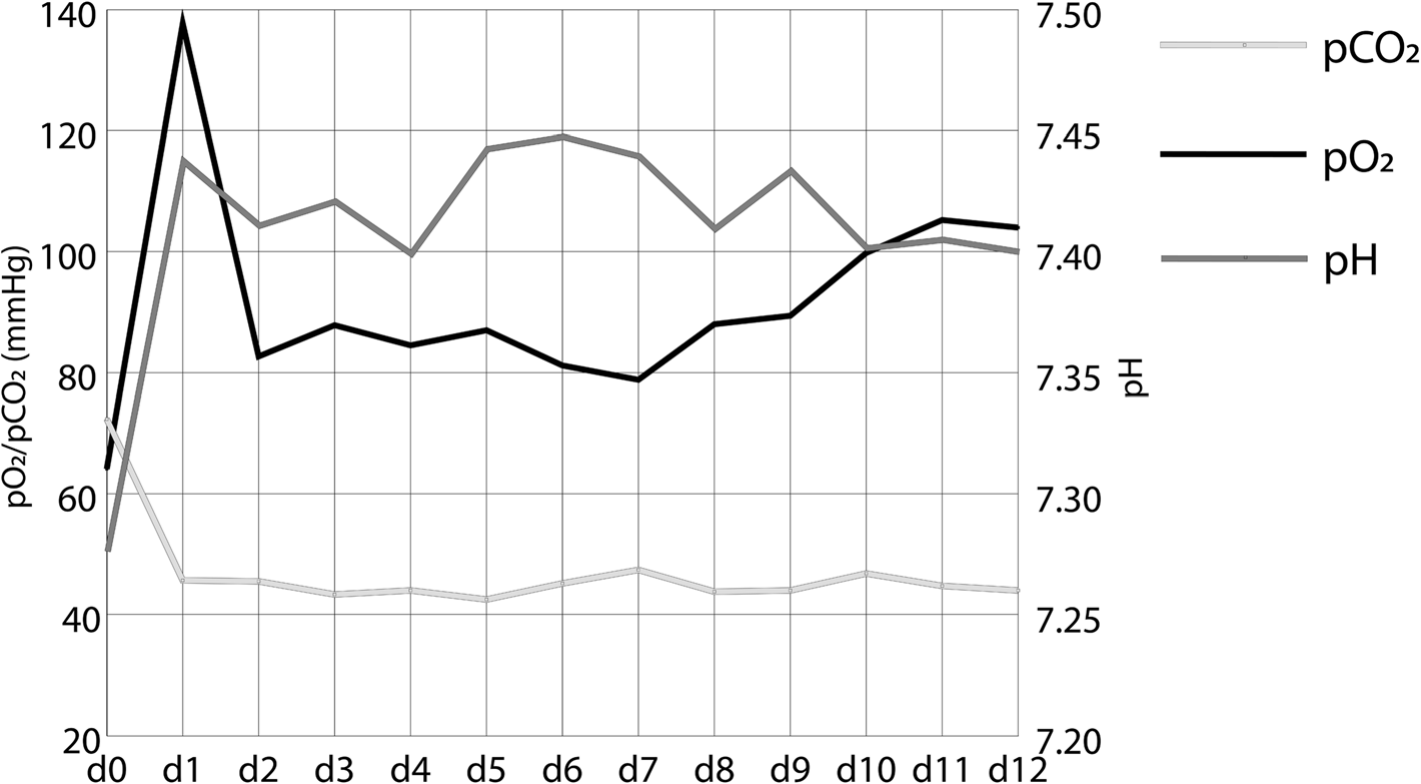
Mean arterial pO_2_, pCO_2_, and pH at onset of extracorporeal membrane oxygenation (ECMO) (day 0) and 12 consecutive days (*n* = 8)

ECMO treatment resulted in a rapid decrease of plateau and driving pressure need with slowly increasing lung compliance over the course of treatment as shown in Fig. 2. Also, it enabled normoxia and normokapnia with a stabilization of the blood pH as shown in Fig. 3.

Mean total duration of ECMO therapy was 388 ± 283 h. Two of the patients received necrectomy and skin grafting under ongoing ECMO therapy with minor bleeding in one of these patients that could be managed by compression therapy.

Patients were administered a median of 19 (10–111) packed red blood cells (PRBCs) and a median of 1 (0–41) platelet concentrates over the course of treatment whereupon all patients received PRBCs but only four patients platelet transfusions.

In one patient, aberrant puncture and cannulation of the femoral artery occurred that needed to be corrected surgically after introducing a perfusion cannula for leg perfusion maintenance and one patient developed thoracic hemorrhage under ECMO therapy that required evacuation of hematoma.

Over the course of treatment, three patients died due to severe sepsis of pulmonary origin with multiple organ failure. Four patients could be successfully weaned from ECMO and discharged, while another patient was transferred to a specialized pulmonary ECMO center, where she could be weaned and discharged shortly after.

### Case report

Patient 4, a 21-year-old male, had the most remarkable clinical course with acute kidney failure and need of renal replacement therapy on day 2 and initiation of V-V-ECMO therapy due to severe ARDS on day 6 after 75% TBSA deep dermal to full-thickness burn and inhalation injury. Prior, prone positioning and inhalative NO therapy had not been successful in improving respiratory function.

After successful ECMO weaning and removal on day 19, the patient developed severe Candida sepsis with respiratory insufficiency and a PO2/FiO2 of 59 mmHg and pH of 6.99 and required emergency recannulation for veno-venous ECMO therapy on day 40 after admission. Albeit, exhausting the settings of the ECMO with maximum blood and oxygen flow, the patient still showed systemic hypoxia, which we attributed to the hyperdynamic septic circulatory situation, where ECMO blood flow relative to the cardiac output is insufficient, a phenomenon described in the literature [17].

For this reason, we decided to insert an additional arterial outflow cannula in the brachiocephalic trunk to oxygenate the brain, resulting in a V-V-A-ECMO setup (Fig. 1b). Afterwards, we saw a rapid decline in lactate as marker of ischemia and later an improvement in systemic oxygenation.

The patient could be weaned from ECMO successfully by day 62 and later discharged to a rehabilitation facility after 172 days of inpatient treatment.

## Discussion

In our cohort of adult burn patients receiving ECMO therapy for ARDS, we saw a hospital mortality of 37.5% with the survival of five out of eight patients with a mean burn TBSA of 37%. None of the deaths were associated to ECMO treatment from our understanding. All but one patient fulfilled the Berlin criteria for diagnosis of severe ARDS, which has been described to result in a mortality of 59.7% in burn patients, while this score does not consider individual severity of burn injury [2].

ECMO treatment requires anticoagulation to prevent clot formation in the tubing and oxygenator of the ECMO device. We use a rather restrictive anticoagulation regime with heparin and a target partial thromboplastin time of 40–50 s and try to postpone operative interventions after successful ECMO weaning where possible. In our presented cohort, two patients received surgical debridement and skin grafting under ongoing ECMO therapy without complications.

We monitor levels of antithrombin III (ATIII) regularly and hold them in the reference range (80–100%) to enable heparin action and avoid clot formation. Importantly, this protocol is in accordance with the recommendations of Martucci et al. in their study on anticoagulation during ECMO therapy [18].

All patients required the administration of more than two PRBCs during their treatment, and while sepsis, disseminated intravascular coagulation (DIC), and bone marrow depression occurred in a number of patients, it can be assumed that the high need for blood cell substitution can be mostly attributed to ECMO therapy. Yet, no life-threatening bleeding complications occurred in our small cohort of patients. In one patient, cannulation complications in the form of an aberrant cannulation of the femoral artery occurred that could be resolved. Overall cannulation complications occur in around 10% of ECMO cases at our institution. In a systematic review of complications in 1042 patients who had received V-V-ECMO for ARDS treatment, bleeding complications were the most common with 29.3% while mortality due to complications occurred in only 6.9% of patients [19].

In the recent literature, Eldredge et al. report a very low hospital mortality of 12.5 % in eight patients with burn injury receiving ECMO for severe ARDS. This study included six pediatric patients and two adult patients with a maximum age of 24 years. Thus, it is not comparable to outcome studies on adult patients but, however, still supports the potential benefit of ECMO treatment [11].

Ainsworth et al. present hospital mortality of 43% in their cohort of 14 patients with burn injury, toxic epidermal necrolysis (TEN), and inhalation injury with severe ARDS receiving ECMO. Excluding two patients with TEN and one patient with inhalation injury only, the hospital mortality of 11 burn patients with a mean burn TBSA of 27% was 54% [12].

A retrospective analysis of 58 burn patients who were registered at the ELSO international registry and who received ECMO therapy from 1999 to 2015 resulted in a hospital mortality rate of 57% and thus similar to the results of 10.601 patients with respiratory failure who were registered in the same registry from 1989 through 2016 [9, 20].

Most cohort studies cover a long period of time as the use of ECMO in burn patients is a rare event. We happened to have a number of burn patients qualifying for ECMO treatment in a rather short period of time so that the reported eight patients were treated in a time interval of only 2 years. This generated a rapid learning curve of the personnel treating these patients and along with the high volume of general ECMO applications at our institution (> 90/year) potentially resulted in improvement of care and the low mortality of the patients.

The conventional ventilatory support versus extracorporeal membrane oxygenation for severe adult respiratory failure (CESAR) randomized control trial was released in 2009 and reported an improvement in the 6-month survival of severe ARDS using ECMO and concluded consideration of ECMO in patients, whose Murray score is 3 or higher and who were not ventilated longer than 7 days with harmfully high pressure settings [21].

This study was criticized, mainly for the lacking standardization of the control group and the lack of a crossover option for the patients of the control group.

In July 2018, the results of the ECMO to Rescue Lung Injury in Severe ARDS (EOLIA) randomized control trial on patients with severe ARDS were presented that was intended to overcome the flaws of the CESAR trial.

The 60-day mortality (and also hospital mortality) in the ECMO group was 35% versus 46% in the control group with a *p* = 0.07. Noteworthy is a crossover of 28% of the control group receiving emergency onset of ECMO and achieving survival of 43% in this subpopulation [8].

While the authors conclude that there is no statistical significance, the study is discussed as being underpowered to demonstrate significance of the shown survival benefit of ECMO therapy. Taking the survival of the crossover patients into account, statistical significance for a survival benefit of ECMO therapy can easily be assumed. Additionally, a reduction of mechanical power applied to the lung via ventilation by 66% could be achieved by ECMO therapy which could be relevant for long-term pulmonary morbidity and mortality beyond the scope of the study [22].

As a possible conclusion of the EOLIA trial, onset of ECMO therapy in patients with severe ARDS refractory to conventional therapy should be aimed for as a survival benefit versus onset of ECMO as an emergency last resort option can be assumed [23]. The latest meta-analysis on the use of ECMO in ARDS including the EOLIA trial concludes reduced 60-day mortality for ECMO therapy [24].

Our results in the small sample of eight burn patients with a hospital mortality of 37.5% are in the range of the results of the ECMO group in the EOLIA trial on not burn-associated severe ARDS. Also, the data of the ELSO registry indicates mortality of burn patients with ARDS receiving ECMO being in the range of total ARDS patients receiving ECMO therapy with 57% [9, 20]. This suggests that the application of ECMO in burn patients with ARDS does not necessarily require different criteria than in other patients with ARDS and similar survival rates can be achieved.

An additional factor in burn patients that should be considered is the need for arterial oxygen tension to provide tissue oxygenation and enable wound healing. It has been shown that wound regeneration highly depends on sufficient tissue oxygenation, which in turn relies on multiple factors with arterial oxygen partial pressure being one of the most relevant [25]. It therefore can be argued that burn patients with large wound surface areas and severe ARDS with hypoxia may have additional benefit from ECMO therapy due to the elevation of arterial oxygen partial pressure and thus potentially improved wound regeneration. Another positive side effect of ECMO treatment in burn patients is the possibility of quick and effective body temperature control.

At our institution, the decision for initiation of ECMO treatment is made interdisciplinary with anesthesiologists, cardiac surgeons, and perfusionists. Conventional measures such as fluid restriction, high PEEP and low tidal volume ventilation, and prone positioning (if no contraindications like opened abdomen) should have been performed prior to initiation of ECMO without significant improvement and the Murray score should be 3 or higher. ECMO therapy should then be initiated without further delay, avoiding structural lung damage due to high pressure ventilator settings.

## Conclusion

ECMO represents an established treatment modality in intensive care medicine with associated risks. As the current studies point to a benefit of an early onset of ECMO in therapy refractory severe ARDS, its use should not be withheld from burn patients with severe ARDS. The results of our patient cohort and of other studies demonstrate encouraging survival rates of ECMO therapy in this subpopulation of patients. As recommended for other patients, we propose transfer of burn patients with severe respiratory insufficiency with unsuccessful conventional treatment and suspected worsening to burn units with the possibility of ECMO treatment to improve outcome.

## Data Availability

The datasets used and analyzed during the current study are available from the corresponding author on reasonable request.

## Abbreviations

ARDS: Acute respiratory distress syndrome
ECMO: Extracorporeal membrane oxygenation
NO: Nitric oxide
PEEP: Positive end-expiratory pressure
TBSA: Total body surface area
